# Detection of *Paraburkholderia* in Clinical Specimens Associated with Use of Nonsterile Ultrasound Gel for Percutaneous Procedures — United States, Canada, and Israel, May 2023⎯April 2025

**DOI:** 10.15585/mmwr.mm7440a2

**Published:** 2025-12-11

**Authors:** Sarah Lim, Paula Snippes Vagnone, Natalie C. Marshall, Christine Lees, Jennifer L. Dale, Becky Smith, Elizabeth Palavecino, Ruth Lynfield, Annastasia Gross, Krista Knowles, Ayelet Michael-Gayego, Violeta Temper, Jacob Strahilevitz, Yonatan Oster, Daniel Grupel, Dan Reshef, Yair Motro, Petrus J. van der Walt, Matthew A. Croxen, Stephanie W. Smith, Bonita Lee, Graham A. Tipples, Bobby Warren, Jacob Moran-Gilad

**Affiliations:** ^1^Minnesota Department of Health; ^2^Public Health Laboratory, Alberta Precision Laboratories, Edmonton, Alberta, Canada; ^3^Division of Diagnostic & Applied Microbiology, Department of Laboratory Medicine & Pathology, Faculty of Medicine & Dentistry, University of Alberta, Edmonton, Alberta, Canada; ^4^Duke University Hospital, Durham, North Carolina; ^5^Wake Forest University School of Medicine, Winston-Salem, North Carolina; ^6^Hadassah-Hebrew University Medical Center, Jerusalem, Israel; ^7^Faculty of Medicine, Hebrew University, Jerusalem, Israel; ^8^Department of Health Policy and Management, Faculty of Health Sciences, Ben Gurion University of the Negev, Beer Sheva, Israel; ^9^Edmonton Base Lab, Alberta Precision Laboratories, Edmonton, Alberta, Canada; ^10^Li Ka Shing Institute of Virology, University of Alberta, Edmonton, Alberta, Canada; ^11^Women & Children's Health Research Institute, University of Alberta, Edmonton, Alberta, Canada; ^12^Infection Prevention and Control, Alberta Health Services, Edmonton, Alberta, Canada; ^13^Department of Medicine, University of Alberta, Edmonton, Alberta, Canada; ^14^Department of Pediatrics, Division of Infectious Diseases, University of Alberta, Edmonton, Alberta, Canada; ^15^Disinfection, Resistance, and Transmission Epidemiology Laboratory, Durham, North Carolina.

**Summary**

**What is already known about this topic?**

Contaminated nonsterile ultrasound gels have been implicated in outbreaks of *Burkholderia* infections associated with improper infection control practices before or during percutaneous procedures.

**What is added by this report?**

During May 2023–April 2025, use of contaminated nonsterile ultrasound gel before percutaneous procedures was associated with detection of genetically related *Paraburkholderia*, an environmental organism not typically associated with human infection, in 42 clinical specimens from the United States, Canada, and Israel. Based on medical record review, one patient had a confirmed invasive *Paraburkholderia* infection.

**What are the implications for public health practice?**

Use of nonsterile ultrasound gel for percutaneous procedures is not recommended. Guidelines on appropriate ultrasound gel use recommend sterile gel for any applications before, during, or after a procedure that breaches the skin at the ultrasound site.

## Abstract

Contaminated nonsterile ultrasound gels have been implicated in outbreaks of *Burkholderia* infections associated with improper infection control practices before or during percutaneous procedures. In August 2024, the Minnesota Department of Health Public Health Laboratory noticed an increase in *Paraburkholderia fungorum* or *Paraburkholderia* species identified from referred clinical isolates. All isolates were recovered from blood cultures, and whole genome sequencing (WGS) confirmed that the isolates were genetically related. Because *P. fungorum* is not an established human pathogen and has rarely been reported in clinical specimens, an investigation was initiated, which was later joined by collaborators in Canada and Israel after similar observations in those countries. Forty-two patients from the United States, Canada, and Israel with genetically linked *P. fungorum* isolated from clinical specimens collected during May 2023–April 2025 were identified. Positive cultures were associated with the use of nonsterile ultrasound gel. Based on medical record review, treating clinicians deemed the isolate a culture contaminant in most cases; one patient had a confirmed invasive *P. fungorum* infection. WGS confirmed the relatedness of isolates from all three countries, including isolates cultured from clinical specimens as well as from nonsterile ultrasound gel products. Review of local practices revealed use of nonsterile ultrasound gel during point-of-care percutaneous procedures, including drawing blood, placing intravenous catheters, and paracentesis. This investigation underscores the continued importance of sterile gel use during percutaneous procedures and highlights the value of collaboration and shared WGS data for the investigation of international outbreaks.

## Investigation and Results

### Identification of *Paraburkholderia* spp. in Blood Cultures

In August 2024, the Minnesota Department of Health Public Health Laboratory (MDH-PHL) noted an increase in blood culture isolates referred from clinical laboratories in Minnesota that were identified as *Paraburkholderia fungorum* or *Paraburkholderia* spp. by 16S rRNA sequencing.^†^ Seven isolates from patients hospitalized from January to August were submitted to MDH-PHL from three laboratories to rule out *Burkholderia mallei* and *Burkholderia pseudomallei*, Tier 1 select agents with bioterrorism potential.^§^ *P. fungorum* is not an established human pathogen and has rarely been reported in clinical specimens (*1*–*3*), and MDH-PHL had not identified *P. fungorum* in any clinical specimens during the preceding decade. Whole genome sequencing (WGS) of the initial seven isolates found that they were closely genetically related. One laboratory reviewed previous test results, and *P. fungorum* was isolated from the blood cultures of eight additional patients who were identified dating back to September 2023. Because of the retrospective nature of the review and the length of time that had passed, isolates from these patients were not available for WGS. Medical record reviews of all 15 patients revealed that in several cases, the treating providers felt the likelihood of clinical infection was low and the positive blood culture represented a culture contaminant. In addition, for each patient, only a single culture bottle tested positive out of one or multiple sets of blood cultures, suggesting possible culture contamination rather than a true infection. This project was reviewed by the Minnesota Department of Health, classified as nonresearch public health surveillance, and was conducted consistent with applicable federal laws.

### Identification of Additional Positive Cultures Through Statewide Review of Laboratory Records

On October 1, 2024, MDH-PHL notified clinical laboratories statewide, calling for a review of laboratory records of any clinical specimens collected after September 2023 with a positive *Paraburkholderia* spp. culture result. An additional 30 positive blood culture results were identified from 30 patients (one isolate per patient), including four from Minnesota, one from North Dakota, and 25 sent to a national clinical reference laboratory for further identification from clinical laboratories in 11 other states. Only the isolate from North Dakota was available for analysis.

Initially, intrinsic contamination of blood culture bottles during manufacture was suspected, because all positive cultures identified until that time involved the same blood culture system manufacturer. Among the 30 positive cultures identified during the statewide review, MDH-PHL was initially able to obtain the isolate from North Dakota for WGS. As the investigation continued into December 2024, MDH-PHL obtained five additional isolates from Minnesota laboratories from patients hospitalized from August to December 2024 that were added to the WGS analysis, for a total of 12 isolates from Minnesota (including the original seven). The additional five isolates exhibited the same genetic relatedness to one another and to the seven original isolates. MDH notified CDC, the Food and Drug Administration (FDA), and the blood culture system manufacturer of these findings.

### Identification of Positive Cultures from North Carolina, Canada, and Israel 

**FIGURE 1:**
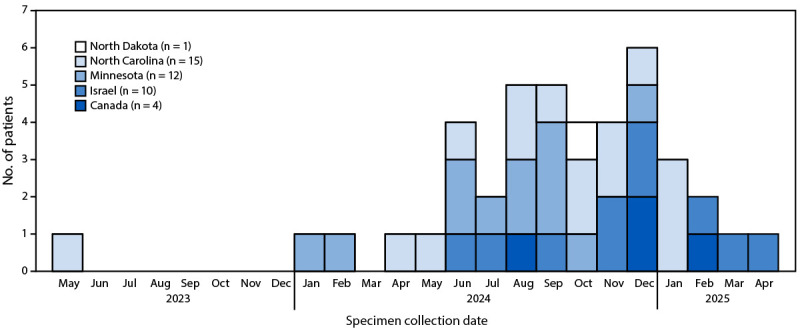
Patients (N = 42) with genetically linked* *Paraburkholderia* spp. isolated from clinical cultures,^†^ by date of initial specimen collection — Minnesota, North Carolina, North Dakota, Canada, and Israel, May 2023–April 2025 * All isolates underwent whole genome sequencing and were found to be genetically related. ^†^ All isolates were recovered from blood cultures, except three isolates from Israel that were recovered from blood culture bottles inoculated with ascitic fluid.

Concurrent with this investigation, *Paraburkholderia* spp. were identified in clinical cultures processed by laboratories in North Carolina (21 cultures), Canada (four), and Israel (10); these three jurisdictions joined the investigation in late 2024 and early 2025. Microbiologists in Canada and Israel reached out to MDH-PHL after noticing the October 1 publicly posted online laboratory alert when searching for information on *P. fungorum*. North Carolina joined the investigation following personal communication between MDH-PHL and laboratory staff members from two clinical laboratories in North Carolina. North Carolina joined the investigation following personal communication between MDH-PHL and laboratory staff members from two clinical laboratories in North Carolina. Among the 21 positive cultures identified in North Carolina, 19 isolates were sent to MDH-PHL (Supplementary Table). These included nine that were identified on the initial list of 25 positive cultures from 11 other states (whose original isolates remained available from the originating laboratory) and sent to the reference laboratory in Minnesota. Among the 19 isolates, 16 were successfully sequenced and added to the WGS analysis, including two isolates from cultures obtained on two different dates from the same patient. Israel and Canada performed WGS on isolates from their jurisdictions and shared results with MDH-PHL. Thus, a total of 43 isolates from 42 patients were included in the WGS analysis (12 from Minnesota, 15 from North Carolina [including one patient with two isolates], one from North Dakota, four from Canada, and 10 from Israel) (Figure 1). All isolates were recovered from blood with the exception of three from Israel, which were recovered from ascitic fluid cultured in blood culture bottles.^¶^

### Identification of Nonsterile Ultrasound Gel as a Potential Contamination Source

Whereas all positive cultures identified by MDH-PHL in the United States until October 1, 2024, involved the same blood culture system manufacturer, isolates from Israel involved a different blood culture system manufacturer, significantly decreasing the likelihood that intrinsic contamination of blood culture bottles with *P. fungorum* was the outbreak source. Investigation of cases in Israel with positive cultures from ascitic fluid obtained via paracentesis identified the use of nonsterile ultrasound gel (ClearImage brand) for point-of-care ultrasound (POCUS) for percutaneous procedures as a potential source of contamination. *P. fungorum* was subsequently cultured in Israel from six containers of the same ultrasound gel product, and WGS of these six isolates demonstrated genetic relatedness to clinical isolates from the United States, Canada, and Israel. Additional cultures from two different brands of nonsterile ultrasound gels in the United States (ClearImage and MediChoice) and Canada (ClearImage) both yielded genetically similar *P. fungorum*, including the same product that grew *P. fungorum* in Israel (ClearImage). In total, *P. fungorum* was isolated from six lots of nonsterile ultrasound gel from two different commercial brands (MediChoice and ClearImage) manufactured by a single company (NEXT Medical Products Company).

### Whole Genome Sequencing

**FIGURE 2:**
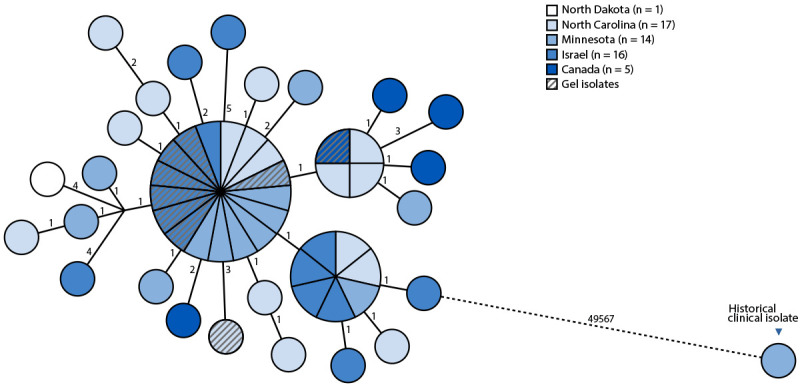
Genetic relatedness of *Paraburkholderia fungorum* isolates using core-genome single nucleotide polymorphism analysis*^,^^†^ — Minnesota, North Carolina, North Dakota, Canada, and Israel, May 2023–April 2025 **Abbreviation**: cgSNP = core-genome single nucleotide polymorphism. * All 52 *P. fungorum* isolates from this cluster (43 clinical isolates [including two isolates from one patient] and nine isolates from ultrasound gel) were analyzed using cgSNP mapping alongside one historical clinical isolate from 2011, generating a 49,606 base cgSNP alignment displayed in a minimum-spanning phylogenetic tree. Labeled distances across connecting lines between nodes indicate the number of cgSNP differences. Nodes are illustrated with sizes proportional to the number of identical genomes. The three larger nodes represent clusters of isolates having zero cgSNP differences. ^†^ Detailed sequencing and bioinformatics methods are available at https://www.health.state.mn.us/diseases/idlab/mmwr.html

In total, WGS was performed on 43 clinical isolates from 42 patients,** nine ultrasound gel isolates (six from Israel, and one each from Canada, Minnesota, and North Carolina), and one historical clinical isolate from 2011.^††^ Sequence data were shared between Canada, Israel, and Minnesota, creating a joint genomic repository for real-time data analysis and cross-validation of results. WGS demonstrated that all clinical and gel isolates were closely related, exhibiting 0–10 single nucleotide polymorphism differences (Figure 2), confirming that all clinical and gel isolates belonged to a single clone of *P. fungorum*. This clone appeared to be very distant from the 2011 historical isolate, implicating contaminated nonsterile ultrasound gel as the source of *P. fungorum*. Further genome analysis demonstrated that the cluster was unrelated to publicly available genomes.

### Characteristics of Patients with Isolates Linked by WGS

Medical records of patients in each jurisdiction were reviewed by members of the investigation team with clinical infectious disease expertise to obtain demographic and clinical characteristics. Where possible, health care providers who had performed an ultrasound-guided percutaneous procedure on the patient were interviewed on their use of ultrasound gel. Among 42 patients with positive cultures for *P. fungorum* genetically related by WGS in the United States, Canada, and Israel during May 2023–April 2025, 39 (93%) had positive blood culture results, the remaining three had positive cultures of ascitic fluid (Table). For one patient, three consecutive blood culture results were positive for *P. fungorum* over several days, and the patient received a diagnosis of a central-line–associated bloodstream infection (CLABSI)^§§^; no other patient had confirmed invasive *P. fungorum* infection based on the medical record review.^¶¶^ Among 37 patients with available clinical information, antibiotic therapy was initiated, modified, or extended for 28 (76%) in response to the positive culture result (e.g., to provide empiric coverage for gram-negative bacteremia or to provide specific coverage for *Paraburkholderia* bacteremia). According to medical record review, providers ultimately deemed *Paraburkholderia* to be a culture contaminant in 25 (68%) cases. In three patients with *P. fungorum*–positive ascitic fluid cultures, peritonitis was ruled out through clinical and laboratory findings. Sixteen (38%) of the 42 patients had a documented ultrasound-guided percutaneous procedure before or during specimen collection, most commonly peripheral intravenous catheter placement and blood collection in an emergency department.*** MediChoice or ClearImage nonsterile ultrasound gel use was confirmed in all facilities except one facility without information, with gel use confirmed for 15 (37%) patients, either through medical record documentation or health care provider interview.

**TABLE:** Characteristics and exposures of patients with genetically linked* *Paraburkholderia* spp. isolated from cultures of clinical specimens^†^ (N = 42) — Minnesota, North Carolina, North Dakota, Canada, and Israel, May 2023–April 2025

Characteristic (no. of patients with available information)	No. (%)
**Age group, yrs (42)**	
<18	10 (23.8)
18–64	18 (42.9)
≥65	14 (33.3)
Median (range)	54 (2 mos–92 yrs)
**Sex (42)**	
Female	27 (64.3)
Male	15 (35.7)
**Jurisdiction (42)**	
Minnesota	12 (28.6)
North Dakota	1 (2.4)
North Carolina	15 (35.7)
Israel	10 (23.8)
Canada	4 (9.5)
**Specimen source (42)**	
Blood	39 (92.9)
Ascitic fluid	3 (7.1)
**Specimen collection location (42)**	
Emergency department	27 (64.3)
Intensive care unit	7 (16.7)
Hospital inpatient	6 (14.3)
Clinic	2 (4.8)
**Number of positive culture bottles (42)**	
Single bottle only	38 (90.5)
Both bottles in single set	3 (7.1)
One or more bottle in multiple sets	1 (2.4)
**Received antibiotics for positive culture result^§^ (37)**	
Yes	28 (75.7)
No	9 (24.3)
**Positive culture ultimately deemed to be contaminant by treating provider^¶^ (37)**	
Yes	25 (67.6)
No	6 (16.2)
Unknown	6 (16.2)
Ultrasound-guided percutaneous procedure performed before or during culture collection** (42)	
Peripheral intravenous catheter placement	8 (19.1)
Paracentesis	3 (7.1)
Venipuncture	4 (9.5)
Other^††^	1 (2.4)
Unknown	26 (61.9)
**Exposure to MediChoice or ClearImage ultrasound gel (41)**	
Patient known to have been exposed^§§^	15 (36.6)
Patient exposure unknown, but product known to have been used at facility	26 (63.4)

* By whole genome sequencing.

^†^ All available isolates underwent whole genome sequencing and were found to be genetically related.

^§^ Includes initiation, modification, or extension of antibiotic therapy in response to the positive culture (e.g., to provide empiric coverage for gram-negative bacteremia or specific coverage for *Paraburkholderia* bacteremia).

^¶^ Includes patients identified during this investigation whose providers were advised of ultrasound gel contamination.

** Procedure documented in medical record as being performed with ultrasound guidance.

^††^ Intracardiac aspiration during resuscitation.

^§§^ Product use documented in medical record or determined during interview to have been used for the patient.

## Public Health Response

Investigation findings were shared with infection prevention professionals at facilities reporting cases, and local practices for the use of ultrasound gel during percutaneous procedures were reviewed. State and local guidance was issued for infection prevention during POCUS procedures and implicated products were removed from those facilities. Information was reported to CDC, FDA, respective health authorities in Canada and Israel, and the gel manufacturer. On May 13, 2025, CDC posted an alert for clinicians describing preliminary findings from this investigation and reinforced its previous recommendation to always use sterile ultrasound gel for percutaneous procedures.

## Discussion

*Paraburkholderia fungorum* (previously *Burkholderia fungorum*)^†††^ is a gram-negative environmental bacterium commonly used as a beneficial microorganism in agriculture to improve crop yields (*4*). This bacterium has rarely been reported in human clinical specimens, and its significance as a pathogen is unclear (*1*–*3*). *P. fungorum* had not previously been reported in association with outbreaks or medical product contamination.

*Burkholderia* species contamination of ultrasound gel and resulting human infection has been previously reported (*5*–*7*). In this cluster, *P. fungorum* was likely introduced into culture specimens or blood during specimen collection, after the application and incomplete removal of contaminated gel from the skin. Culture collection through a peripheral intravenous catheter is also associated with a higher risk for contamination (*8*), although it was not clear for all cases in this investigation whether positive blood cultures represented contamination of specimens at the time of the procedure or true bacteremia resulting from inoculation of the organism into the patient’s bloodstream. Examples of factors suggestive of culture contamination included instances in which patients had no signs or symptoms of infection at the time of the blood culture draw or had a clear alternative diagnosis or pathogen identified that was more consistent with their clinical presentation, but it was not possible to determine on retrospective chart review with certainty in each case whether the positive culture represented true infection. Unlike other outbreaks in which contaminated ultrasound gel has caused invasive infections, this investigation identified only one patient with confirmed invasive *P. fungorum* infection. It remains unclear whether this represents an intrinsic lack of *Paraburkholderia* virulence or whether cases of invasive infection are underreported. Although treating providers ultimately assessed *Paraburkholderia* to be a blood culture contaminant in two thirds (68%) of patients with available information, three fourths (76%) of patients received antibiotics to empirically treat gram-negative bacteremia or to specifically treat *Paraburkholderia* bacteremia. This finding is consistent with previous studies documenting the association between blood culture contamination and unnecessary antibiotic use (*9*) and might also reflect more conservative management because of the high mortality of gram-negative sepsis and the infrequency of gram-negative organisms as contaminants in blood cultures.

The findings from this investigation highlight the need for vigilance regarding best practices for ultrasonography around invasive procedures. Existing guidelines on the use of ultrasound gel are clear that health care personnel should always use only sterile, single-use ultrasound gel products for ultrasonography in preparation for, or during, percutaneous procedures (*10*). Health care personnel who perform percutaneous POCUS procedures, including peripheral intravenous catheter placement and venipuncture, should be trained in the appropriate use of ultrasound gel. If nonsterile gel is inadvertently applied before percutaneous procedures, it should be thoroughly removed from the skin before performing skin antisepsis (*5*).

### Limitations

The findings in this report are subject to at least two limitations. First, despite routine use, ultrasound gel and POCUS for percutaneous procedures (e.g., venipuncture) are often not documented; thus, a retrospective chart review was unable to confirm gel use for all patients. Second, identification of *P. fungorum* in clinical specimens is not reportable; thus, its incidence is likely underestimated.

### Implications for Public Health Practice

Infections and contaminated clinical specimens due to contaminated ultrasound gel continue to occur (*5*–*7*) and might become a larger concern as POCUS use becomes more common in clinical care, especially in emergency and intensive care settings. It is important for health care personnel, health care facilities, infection prevention specialists, and manufacturers to be aware of this risk and consider additional infection prevention measures. For instance, this investigation determined that several facilities reported stocking single-use packets of nonsterile gel; this packaging format itself might have been misinterpreted as a marker of sterility and could have contributed to its inappropriate use for percutaneous procedures. Manufacturers and health care facilities should ensure that nonsterile gel, particularly in single-use packets, is clearly labeled as nonsterile. Quality improvement measures to reduce blood culture contamination, such as those developed by CDC to promote best practices in blood culture collection and reporting of possible skin contaminants, can also reduce the rate of false positive cultures and limit unnecessary antibiotic therapy. The multiple countries involved in this investigation highlight the value of WGS for international surveillance and outbreak response, which allowed rapid sequence data sharing to establish the outbreak scope and source.

## Footnotes

* These authors contributed equally to this report.

† 16S rRNA sequencing is a method used to taxonomically classify cultured bacteria by analyzing sequence information from variable regions in the 16S rRNA gene that is present in all prokaryotes.

§ MDH-PHL is a Laboratory Response Network Reference Laboratory and provides confirmatory testing for Tier 1 agents of potential bioterrorism, including *B. mallei* and *B. pseudomallei*.

¶ Time to culture positivity = 3–5 days.

** One patient in North Carolina had two isolates included in WGS. This patient had a central venous catheter and had three consecutive positive blood cultures for *P. fungorum* over 7 days, followed by a diagnosis of a central line–associated bloodstream infection (CLABSI).

†† WGS data were deposited under the BioProject ID: PRJNA1260949. A summary of short-read raw sequence and assembly data from isolates included in this analysis (including 52 isolates from this investigation, a historical clinical isolate from 2011, and 24 publicly available genome sequences), long-read sequence data (including three isolates), core genome multilocus sequence typing of *P. fungorum* cluster isolates compared with publicly available genomes, and detailed sequencing and bioinformatics methods are available at https://health.state.mn.us/diseases/idlab/mmwr.html.

§§ Same North Carolina patient with CLABSI and two isolates were included in WGS. Cultures were cleared after removal of the catheter.

¶¶ Examples of factors suggestive of contamination in these cases included 1) single positive culture only; 2) no signs or symptoms of infection at time of blood culture draw; 3) clear alternative infectious diagnosis; 4) alternative typical pathogen identified in culture; 5) longer time to culture positivity than that typically observed for an infectious pathogen (≥3 days); and 6) a treating clinician’s determination of blood culture contamination. In many cases, it was not possible to determine with certainty on retrospective chart review whether the positive culture represented true infection.

*** Although POCUS was not documented in the majority of patients’ medical charts, all facilities reported that ultrasound guidance was common practice for many bedside procedures, including peripheral intravenous catheter placement and venipuncture.

††† The *Paraburkholderia* genus was proposed in 2015 as a distinct genus from *Burkholderia* to separate pathogenic species (such as *B. cepacia* complex) from other environmental species not commonly associated with disease.
